# Bone Homeostasis and Gut Microbial-Dependent Signaling Pathways

**DOI:** 10.4014/jmb.2104.04016

**Published:** 2021-06-15

**Authors:** Xiaohui Zhong, Feng Zhang, Xinyao Yin, Hong Cao, Xuesong Wang, Dongsong Liu, Jing Chen, Xue Chen

**Affiliations:** 1 Wuxi School of Medicine, Jiangnan University, Wuxi 214122, P.R. China; 2 Department of Orthopedics, Affiliated Hospital of Jiangnan University, Wuxi 214125, P.R.China; 3 Clinical Assessment Center of Functional Food, Affiliated Hospital of Jiangnan University, Wuxi 214125, P.R. China; 4 Department of Endocrinology, Affiliated Hospital of Jiangnan University, Wuxi 214125, P.R. China; 5 Nutritional Department, Affiliated Hospital of Jiangnan University, Wuxi 214125, P.R. China

**Keywords:** Bone homeostasis, gut microbiota, signaling pathway, bone metabolism

## Abstract

Although research on the osteal signaling pathway has progressed, understanding of gut microbial-dependent signaling pathways for metabolic and immune bone homeostasis remains elusive. In recent years, the study of gut microbiota has shed light on our understanding of bone homeostasis. Here, we review microbiota-mediated gut–bone crosstalk via bone morphogenetic protein/SMADs, Wnt and OPG/receptor activator of nuclear factor-kappa B ligand signaling pathways in direct (translocation) and indirect (metabolite) manners. The mechanisms underlying gut microbiota involvement in these signaling pathways are relevant in immune responses, secretion of hormones, fate of osteoblasts and osteoclasts and absorption of calcium. Collectively, we propose a signaling network for maintaining a dynamic homeostasis between the skeletal system and the gut ecosystem. Additionally, the role of gut microbial improvement by dietary intervention in osteal signaling pathways has also been elucidated. This review provides unique resources from the gut microbial perspective for the discovery of new strategies for further improving treatment of bone diseases by increasing the abundance of targeted gut microbiota.

## Introduction

With the lifespan extension, population aging has become a global problem [1]. Aging affects skeletal system, and decreases in bone mineral density (BMD) are progressively caused by aging [2]. The treatment and care of the related complications of bone diseases are typically associated with a heavy socioeconomic burden [3]. Therefore, restoring metabolic and immune bone homeostasis has become a significant concern [4]. In recent years, the osteal molecular signaling pathway has been reported connected with the abundance and diversity of gut microbiota, although most of the underlying mechanisms are unclear [5]. The intestinal microenvironment can affect the bone microenvironment both directly and indirectly. The risk of suffering bone diseases is known to increase as a consequence of intestinal dysbiosis related to undernutrition [6], drugs [7], psychosocial stress [8], and viral infection [9]. In this review, we demonstrate fundamental BMP, WNT and RANKL signaling pathways mediated by metabolites and translocation of gut microbiota, which are one of mechanisms in the bone homeostasis. We further summarize the key processes, including immune responses, secretion of hormones, fate of osteoblasts and osteoclasts and absorption of calcium, in mechanisms underlying gut microbiota involvement in these signaling pathways. Remarkably, we also discuss the promotion of skeletogenesis by the change of dietary habits, and the use of probiotics, prebiotics and synbiotics, which can protect bone by increasing the abundances of specific gut bacteria. In the future, restoring bone homeostasis via gut microbial-dependent signaling pathways may be an attractive therapeutic strategy to combat bone diseases.

## Osteal Homeostasis Signaling Pathways

Bone has self-healing and regenerative abilities and contains three types of cells in adults, including osteoblasts and osteocytes from mesenchymal stem cells and osteoclasts from haematopoietic cells in bone marrow. There are two key cell types that play an important role in bone homeostasis: osteoclasts that absorb bone matrix and osteoblasts that synthesise bone matrix. Osteocytes promote the balance of osteoblasts and osteoclasts in bone construction and bone remodeling, and maintain bone homeostasis by ensuring the balance of calcium and phosphorus metabolism [10]. Bone homeostasis is related to cell development and activation as well as mediation of metabolic and immune activities via signaling pathways. Recent research has shown that the BMP/SMADs, Wnt/β-catenin and OPG/RANKL/RANK signaling pathways play primary roles in bone homeostasis [11, 12]. Bone formation depends on BMP and Wnt induced anabolism, and bone resorption depends on RANKL induced catabolism. Moreover, skeletal dysplasia may be due to abnormal signaling, which results from excessive enhancement or reduction of the expression of transcription factors related to bone homeostasis.

### Signaling Pathways in Bone Homeostasis

BMP, as a ligand, binds to the receptor SMADs. Activated SMAD complexes are able to regulate gene expression in osteoblasts and osteoclasts by recruitment of chromatin-remodelling machinery and specific transcription factors (*e.g.*, *RUNX2*) [13]. The intensity and duration of BMP signaling pathway are strictly regulated at various levels via intra-or extracellular mechanisms, which are critical for the progression and maturation of osteogenesis [14]. Typical BMP pathway effectors are SMAD1, SMAD5 and SMAD8, and the weaker expression of SMADs in osteoblasts leads to a lower of osteogenic rate. The BMP/SMADs signaling pathway affects most tissue types of the skeletal system, ameliorating diseases of skeletal overgrowth and repairing damage to bone and joints [15]. Accordingly, recombinant human BMPs have been developed as clinical agents to repair bone defects [16].

Another osteogenesis pathway, the canonical Wnt/β-catenin signaling pathway is initiated by binding of the Wnt ligand to a double receptor complex comprised of frizzled (FZD) and either LRP5 or LRP6 [17]. Mouse genetic studies have confirmed the importance of canonical Wnt signaling in the regulation of bone homeostasis, with activation of the pathway leading to increased bone mass and strength, whereas pathway inhibition has the opposite effect. This pathway is now the target for therapeutic intervention to restore bone strength in millions of patients at risk from bone fracture [18, 19]. Wnt/β-catenin signaling inhibits the differentiation of mesenchymal stem cells into chondrocytes and adipocytes and strengthens differentiation into osteoblasts. For example, conditional Wnt1 expression in osteoblasts promoted rapid increases in bone mass by rapidly expanding osteoblast numbers [20]. T lymphocytes are known to secrete Wnt10b, and bone formation is dependent on permissive effects on T cells involving Wnt10b induced bone anabolism [21]. Wnt10b-induced osteoblasts regulate bone anabolism of PTH to promote bone formation and strengthening osteoblast differentiation [22]. Additionally, under inflammatory conditions, lymphocytes secrete TNF-α and RANKL, driving up bone resorption and loss of BMD [21].

Different from the two pathways aforementioned, the RANKL signaling system is the physiological master regulator of osteoclast recycle and bone resorption [23]. It is also known as TNF-related activation induced cytokine, osteoprotegerin ligand or osteoclast differentiation factor. RANKL pathway promotion of bone resorption may be involved in three pathways [24], namely the NF-κB, c-jun amino terminal kinase and protein kinase B pathways. RANKL signaling pathway is negatively regulated by OPG [25]. RANKL, RANK and OPG are also participate in the immune responses related to bone metabolism. RANKL activates osteoclastogenesis, whereas OPG inhibits it [26]. Mutations in the RANKL gene result in human autosomal recessive osteonecrosis and loss of osteoclasts [27]. RANKL directly interacts with the gut epithelium to control CCL20 expression and microfold cell differentiation, which results in a decrease in microbial diversity and unbalanced immune homeostasis [28]. In murine models, deficiency of sex steroids increases gut permeability and upregulates RANKL expression in the small intestine and the bone marrow [29].

### Osteal Signaling Pathway Network

 BMP, Wnt, OPG and RANKL are critical molecules that regulate the effects of mechanical loading on bone formation [30]. Mechanical instability is known to downregulate *BMP2* and *WNT16* mRNA levels, which can be rescued by inhibition of glycogen synthase kinase-3β (GSK-3β). Furthermore, enhanced expression of OPG mRNA has been correlated to decreased osteoclast numbers, and OPG secretion from osteoblasts is regulated by Wnt/β-catenin signaling [31].

Thus, the Wnt and the OPG/RANKL/RANK signaling pathways can act as key mediators of bone homeostasis and interact with each other in bone remodeling [32]. Activating Wnt/β-catenin signaling by inhibiting GSK-3β would therefore reduce instability-induced bone loss. BMP, Wnt and OPG engage in synergistic roles for bone protection and osteogenesis in the bone metabolic signaling network to a great extent, whereas RANKL has an antagonistic role. The cooperation of BMP, Wnt and OPG has a more powerful effect on bone metabolism than the independent action of these proteins. BMP-inactivated stem cells also exhibit up-regulated expression of the Wnt7a, Wnt7b and Wnt16 ligands and Frizzled-10 receptor. A previously unknown intra-stem cell antagonistic competition between BMP and Wnt signaling has also been shown to regulate the differentiation of stem cells into osteoblasts [33]. The RANK–RANKL–OPG catabolic signaling pathways are co-regulated via PTH, whereas the anabolic Wnt signaling pathway is regulated via competitively binding reactions between Wnt and the Wnt receptors LRP5/6 [34]. Taken together, the interaction of the BMP, Wnt and OPG signaling pathway depends on the microenvironment at multiple levels. The in-depth study of this signaling pathway network may provide molecular-based strategies for treating metabolic bone diseases.

## Gut Microbiota

Gut microbiota have attracted increasing attention over the past 15 years [35]. Trillions of microorganisms coexist with the human body, and more than 150,000 microbial genomes have been found through the assembly of metagenomes of approximately 5,000 species [36]. The gut microbiota has different functions that participate in the regulation of host metabolism and immunity; however, research of gut microbiota is still in its infancy [37]. More than 1000 different microorganisms compose the gut microbiota, of which approximately two-thirds are unique to each individual [38]. The overall gut microbial structure is remarkably stable over time with *Bacteroides*, *Prevotella* and *Faecalibacterium* remaining the three most abundant taxa in a healthy population [39]. Changes in the gut microbiota are related to a variety of chronic diseases, such as diabetes [40], obesity [40], inflammatory bowel disease [41], malnutrition [42, 43], arthritis [44] and osteoporosis [45], and the gut microbiota may also act as a mediating factor that can affect metabolic diseases [46, 47]. The gut microbiota acting through microbiota-derived metabolites and microbial translocation can communicate locally and systematically with the host. Thus, alterations in gut microbial composition can improve mineral absorption and skeletal health [48]. Furthermore, bone homeostasis is expected to be restored via the gut microbial-dependent signaling network.

## Microbiota-Based Gut–Bone Crosstalk

### Gut Microbial Metabolites

The gut microbiota extracts energy from food for the host, boosts epithelial growth and excludes pathogen colonisation. In addition, gut microbiota metabolites are crucial for the maturation of the immune system and cell protection. These metabolites explain the anatomically distant biological effects of gut microbiota and can influence bone homeostasis via a molecular signaling network, which includes short-chain fatty acids (SCFA), trimethylamine N-oxide (TMAO) and microbe-associated molecular patterns (MAMPs) [49, 50]. In addition, gut microbiota metabolites from phytoestrogens and dietary plant polyphenols promote bone metabolism. However, the study of osteal signaling pathways mediated by gut microbiota metabolites requires further study.

### SCFAs May Promote Bone Homeostasis

**Fig. 1:**
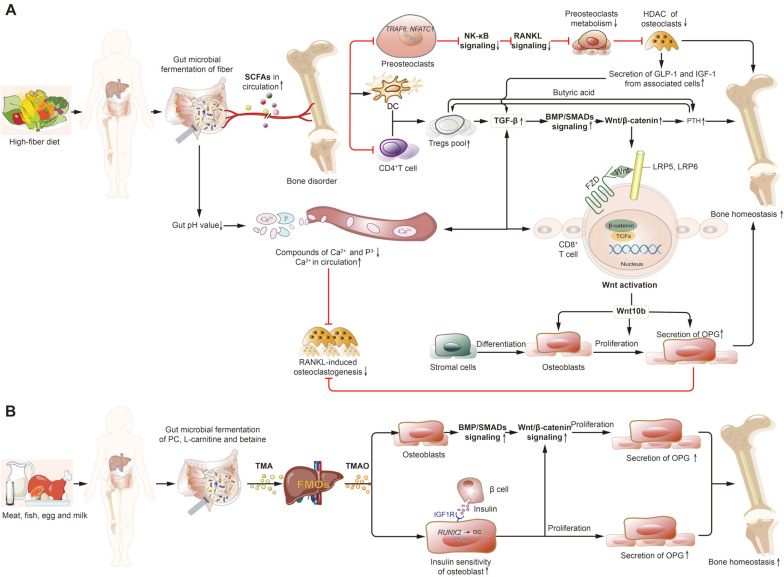
Gut microbial metabolites in osteal signaling pathway. (**A**) SCFAs promote bone homeostasis. SCFAs can inhibit RANKL signaling, upregulate TGFβ-BMP-Wnt signaling, and promote the absorption of calcium by lowering the pH in the intestinal tract. (**B**) TMAO promotes bone homeostasis. TMAO promotes proliferation of osteoblasts by upregulating BMP-Wnt signaling and improving insulin sensitivity. Abbreviations: SCFA, short-chain fatty acid; HDAC, histone deacetylase; DC, dendritic cell; TGF-β, transforming growth factor-β; FZD, frizzled; LRP5, low-density lipoprotein receptor-related protein 5; TCFs, T cell factors; IGF-1, insulin-like growth factor-1; GLP-1, glucagon-like peptide-1; PTH, parathyroid hormone; PC, phosphatidyl choline; TMA, trimethylamine; TMAO, trimethylamine N-oxide; OC, osteocalcin.

SCFAs are produced by gut microbial fermentation of dietary fibre and include acetic acid, propionic acid and butyric acid, which provide nutrition for intestinal epithelial cells and inhibit the inflammatory response [51, 52]. Specifically, Firmicutes produce high amounts of butyrate, whereas Bacteroidetes, including *Bacteroides acidifaciens*, produce high levels of acetate and propionate [53]. SCFAs regulate insulin-like growth factor-1 (IGF-1) and glucagon-like peptide-1 (GLP-1) via inhibition of histone deacetylase (HDAC) and activating receptor γ as a G-protein-coupled receptor ligand and peroxisome proliferator to indirectly promote bone formation as the signal molecules [36, 54]. HDACs can remove acetyl groups from lysine side chains in histones and disturb cellular processes including gene transcription, DNA repair, and cytokine signaling cascades (*e.g.*, TGF-β-BMP-SMADs). Thus, inhibition of HDACs can facilitate the intramembranous and endochondral ossification, as well as bone resorption [55]. Butyric acid can not only inhibit HDAC in osteoclasts but also directly induce metabolic reprogramming of osteoclast precursors, promote glycolysis at the cost of oxidative phosphorylation and downregulate expression of pivotal osteoclast genes, including *TRAF6* and *NFATC1*, to SCFA promotes bone homeostasis mediated by gut microbial-dependent signaling pathway as described in Fig. 1A. Besides, SCFAs also promote bone formation by lowering the pH in the intestinal tract and reducing the formation of calcium and phosphorus complexes, supporting the free calcium ions can be easily absorbed into the blood circulation [56]. Calcium can active Wnt signaling and inhibit RANKL-induced osteoclastogenesis. Butyric acid expands the intestinal absorption area and facilitates the absorption of calcium. PTH-dependent bone formation requires butyric acid production by gut microbiota [57]. In addition, the indirect effect of SCFAs may explain their ability to induce activation of Wnt-dependent osteogenesis by regulatory T cells (Tregs). Tregs inhibit osteoclast differentiation through secreted anti-osteoclast factors, cell–cell contact dependence and indoleamine 2, 3-dioxygenase-induced mechanisms.

Besides, Wnt signaling pathway can be regulated by gut microbiota to promote hematopoietic stem cell self-renewal, which is due to the association between gut dysbiosis and hematological abnormalities in both humans and mice [58, 59]. On the one hand, SCFAs can directly promote hematopoiesis after being transported to the bone marrow and act as signaling molecules on peripheral tissues. Treatment with SCFAs, especially propionate, rendered mice substantially resistant to DNA damage and reactive oxygen species release in hematopoietic tissues. Significantly, Lachnospiraceae and Enterococcaceae as the SCFAs-producing bacteria play protective roles in promoting hematopoiesis [60]. On the other hand, gut microbiota supports post-transplant hematopoietic reconstitution through its role in dietary energy uptake [61].

### TMAO May Promote Bone Homeostasis

The gut microbiota transform phosphatidyl choline, L-carnitine and betaine in diet (meat, fish, egg and milk) into trimethylamine, and further converts trimethylamine to TMAO by hepatic flavin monooxygenases in liver [62]. In particular, serum TMAO is positively correlated with the abundance of certain gut microbiota in humans, including the *Prevotella*ceae, *Enterobacter*iaceae, Ruminococcaceae, and Clostridiaceae [63]. Several TMA-producing commensals have been identified, such as *Proteus mirabilis*, *Escherichia fergusonii*, and *Proteus penneri*. TMAO may protect against BMD reduction during weight loss [64]. A greater reduction in plasma levels of TMAO is associated with a greater loss in whole-body BMD, independent of body-weight changes. Gut microbiota-dependent metabolite TMAO improved insulin sensitivity and glucose metabolism during weight-loss intervention for obese patients [65]. Insulin is a potent stimulator of osteoblast differentiation and osteocalcin gene expression [66]. Insulin promotes proliferation of osteoblasts by interacting with the IGF-1 receptor that is present on osteoblasts in bone, and promotes secretion of osteocalcin by enhancing expression of osteoblast genes, resulting in higher BMD [67, 68]. TMAO upregulates the expression of osteoblast genes including *RUNX2* and *BMP2*, which mainly affect bone formation by the BMP/SMADs and Wnt signaling pathways, suggesting that TMAO promotes osteogenic differentiation and improves bone quality [69]. TMAO promotes bone homeostasis mediated by gut microbial-dependent signaling pathway as described in Fig. 1B. However, TMAO is increased by insulin resistance and associated with several sequelae of metabolic syndrome in humans [70], and higher TMAO levels lead to a decreased gut microbiota diversity and an increased risk of atherosclerosis [71], indicating that further research is needed to maximise the therapeutic benefits.

### Gut Microbial Translocation

Translocation of gut microbiota may induce and aggravate inflammatory bone diseases. Low-BMD individuals have a smaller number of operational taxonomic units and bacterial taxa at each level. Furthermore, functional prediction revealed that 93 metabolic pathways were significantly different between low-BMD and high-BMD individuals [72]. In addition, suppression of gut microbiota translocation ameliorates vascular calcification through inhibition of toll-like receptor (TLR) 9-mediated BMP-2 expression [73], which ameliorates inflammatory bone diseases via suppression of the TLR9/NF-κB/BMP-2 signaling pathway. Translocations of dead or living bacteria or fungi from mucosa to joints could contribute to the onset and flares of inflammatory rheumatisms [74]. Additionally, changes in the colonisation time and quantity of gut microbiota in different gut locations will affect bone resorption and bone mass [75, 76]. Translocation of gut microbiota has the potential to serve as a biomarker of bone metabolic activity as well as a target for therapies to improve bone structure and quality [77].

## Gut–Bone Holistic Signaling Network Mediated by Gut Microbiota

Enteric dysbiosis destroys bone homeostasis by inhibiting osteoblasts and activating osteoclasts. The gut microbiota can have an important impact on the natural process of diseases but the mechanism remains elusive, especially the impact on bone [78]. The gut microbiota plays a crucial role in the osteal signaling network by shaping the immune response and determining the fate of osteoclasts and osteoblasts and affecting hormone secretion and calcium absorption.

### Roles of Immune Response Mediated by Gut Microbiota in the Osteal Signaling Network 

Metabolic bone abnormality may be caused by immune disorders mediated by gut microbota. For one thing, gut dysbiosis and direct contact between the gut microbiota and immune cells stimulate the immune response of bone tissue [52]. For instance, transplanting gut microbiota from donors with inflammatory bowel disease into germ-free mice, compared with transplantation of gut microbiota of healthy donors, increased the numbers of intestinal Th17 and Th2 cells and decreased those of retinoid-related orphan receptor gamma-t^+^ (RORγt^+^) iTreg cells [79]. The development of RORγt^+^ iTreg cells is regulated by specific transcription factors RORγt, and generation of RORγt^+^ iTreg cells take advantage of microbiota-derived material acquired by dendritic cells from the gut lumen[80]. Tregs produce strong inhibitors of osteoclastogenesis, such as IL-10, IL-4, OPG or cytotoxic T lymphocyte protein 4, which counterbalance the RANKL signaling pathway [81]. Interestingly, both natural regulatory Tregs and TGFβ-induced CD4^+^ Foxp3^+^ Tregs (iTregs) can suppress osteoclastogenesis, but only iTregs sustained this effect in the presence of IL-6 and dramatically decreased NF-κB levels in osteoclasts [82]. Therefore, iTregs may be therapeutically beneficial in related bone diseases via inhibition of the RANKL signaling pathway.

For another, the gut microbiota affects bone immunity through bacterial components and bacterial products. The key players mediating the communication between host and microbes are pattern recognition receptors (PRRs), which are expressed by innate immune cells such as dendritic cells [83]. MAMPs, such as lipopolysaccharides (LPS) and flagellin, are introduced into systemic circulation, and recognised by PRRs in bone tissue [84]. After MAMPs enter bone tissue, they can stimulate immune cells to release inflammatory cytokines and subsequently, inflammation occurs in local bone tissue [85]. For example, in a mice model of OA caused by instability of the medial meniscus, LPS produced by *Proteus* spp. enters the circulatory system, promoting autoimmunity and causing low degree inflammation in bones and joints [86]. MAMPs are known to directly affect bone remodelling by stimulating innate immune receptors on bone cells, such as PRRs and TLRs [87, 88]. TLR-mediated inflammation can result in osteoclastic bone erosion by interconnecting the myeloid stromal cell and Th17 cell in joint vascularization [89].

### Roles of Hormones Mediated by Gut Microbiota in the Osteal Signaling Network

The secretion of glucose-dependent incretin and sex hormone are influenced by gut microbiota. The beneficial gut bacteria can stimulate intestinal cells to secrete incretin, including GIP-1 and GLP-1 [90]. The binding of GIP-1 to the osteoblast surface receptor increases the expression of type I collagen, promotes the maturation and mineralisation of collagen matrix, increases the activity of alkaline phosphatase, promotes the secretion of TGFβ, and upregulates Wnt and TGFβ/BMP signaling pathways, thereby promoting bone formation [91]. GLP-1 stimulates bone formation and inhibits bone absorption via promotion of insulin secretion by β-islet cells and calcitonin by thyroid C cells. Women are prone to osteoporosis due to decreases in levels of sex hormones [92], whereas the BMD of athletes with amenorrhea improved after administering estradiol [93]. The female athlete triad is a condition seen in physically active female athletes, and consists of low energy availability, menstrual dysfunction and BMD [94]. These athletes may have low energy due to lack of energy from the diet metabolised by gut microbiota; meanwhile, oestrogen deficiency and bone loss may be related to gut microbial imbalance. Treating sex steroid-deficient mice with the probiotic *Lactobacillus rhamnosus* GG reduced gut permeability, dampened inflammation of intestinal and bone marrow and protected against bone loss [29]; similarly, probiotics prevented bone loss in ovariectomized mice [95, 96]. However, hormones can also affect gut microbiota. For example, oral testosterone treatment can reduce the overall abundance of gut microbiota and increase the abundance of Firmicutes and *Bacteroides* spp. [97].

### Roles of Osteoclasts and Osteoblasts Mediated by Gut Microbiota in the Osteal Signaling Network

Dysbiosis of gut microbiota can lead to osteogenesis disorder. For instance, the trabecular volume and BMD of chicken foetal phalanges are decreased by injecting chicken embryos with dysbiosis-derived LPS [75], with inhibition of the expression of osteoblast genes (*OCN*, *RUNX2*, *OSX*, and *DLX5*). Maladjusted gut microbiota interferes and ultimately limits embryonic ossification by activating the NF-κB signaling pathway, stimulating the release of IL-6 and TNF-α, activating retinoic acid signaling and directly inhibiting the transcription of *DLX5*. Imbalance of gut microbiota in mice with a high-fat diet leads to increase in the abundance of *Verruciformes*, *Actinomycetes* and *Proteus* spp. in the ileum and cecum [98]. The destruction of bone microenvironment by increasing LPS levels promotes the differentiation of haematopoietic stem cells into adipocytes. The decrease in *RUNX2* expression strongly inhibits bone marrow niche genes (*JAG1*, *CXCL12*, and *IL7*), resulting in decreased osteoblast numbers and inhibition of bone formation. In addition, the level of intestinal butyrate is decreased by disrupting the composition and reducing the amount of gut microbiota in mice [22, 45, 57], which ultimately shortens bone lifespan by inhibition of the differentiation and proliferation of osteoblasts. The above results indicate that dysbiosis can lead to obstacles to osteogenesis.

### Roles of Calcium Mediated by Gut Microbiota in the Osteal Signaling Network

Calcium is an essential nutrient for bone mineral deposition. The gut microbiota improves bone metabolism by increasing calcium absorption and ultimately reducing the risk of osteoporosis and fractures. Calcium absorption positively correlated with increases in faecal microbial community diversity [99]. For instance, the abundance of gut *Bifidobacterium* and *Clostridium* spp. and unclassified Clostridiaceae and Firmicutes members increases calcium absorption. A strategy for improving calcium absorption is enhancing intake of prebiotic dietary fibres, such as nondigestible oligosaccharides and polysaccharides. The explanation for the underlying mechanism of prebiotic-induced calcium absorption is that gut microbial production of SCFAs by fermentation provides an acidic environment ideal for increasing the solubility and transcellular absorption of calcium [100]. This fermentation may directly increase calcium absorption through hydrogen ion exchange or indirectly through hypertrophy of the intestinal mucosa to increase the surface area for greater mineral diffusion [101]. Calcium also modulates the gut microbiota in a prebiotic manner, establishing a gut–bone crosstalk and promoting a healthier metabolic profile [102]. Calcium influx can activate the Wnt/β-catenin and OPG signaling pathways (Fig. 1A). For example, the duck egg white-derived peptide VSEE (Val-Ser-Glu-Glu) is metabolised by gut microbiota and regulates bone metabolism by the Wnt/β-catenin signaling pathway [103]. VSEE promotes differentiation of pre-osteoblasts, which is attributed to stimulation of calcium influx, and then to activation of the Wnt/β-catenin and OPG signaling pathways. Additionally, materials coupled with calcium-binding BMP-2 mimicking peptides can promote bone regeneration [104, 105]. Activin A receptor type 1-mediated BMP signaling regulates RANKL-induced osteoclastogenesis via the canonical BMP/Smad signaling pathway [106]. Thus, RANKL-induced calcium oscillations are important trigger signals for osteoclastogenesis [107].

### An Osteal Signaling Network Mediated by Gut Microbiota

As mentioned above, BMP, Wnt and OPG/RANKL are the main molecules that form a signaling pathway network believed to regulate bone homeostasis. Wnt/β-catenin promotes the secretion of OPG by osteoblasts. Enhanced expression of OPG-related genes reduces the number of osteoclasts; BMP plays a synergistic role in this process and RANKL has an antagonistic role. The gut microbiota participates in the bone metabolic signaling pathway network through translocation and generation of metabolites. For example, SCFAs produced by gut microbiota promote bone metabolism by enhancing the BMP, Wnt and OPG signaling pathways and inhibiting the RANKL signaling pathway [36, 55] (Fig. 1A). TMAO mainly affects bone formation by upregulating BMP/SMADs and Wnt signaling pathways (Fig. 1B). However, upregulating the BMP signaling pathway can repair damaged bone tissue by decreasing secretion of MAMPs. Furthermore, the aforementioned gut microbiota mechanism regulates bone metabolism and immunity by impacting the immune response, inducing the secretion of hormones, influencing the fate of osteoclasts and osteoblasts and affecting calcium absorption. These actions are closely related to the main signaling pathway networks for bone homeostasis as described below.

First, the BMP/SMADs signaling pathway may restore bone homeostasis via the gut microbial-dependent signaling network in a positive manner. Immune cells regulate bone homeostasis by releasing pro-inflammatory and anti-inflammatory cytokines and utilising gut microbiota metabolites. These substances upregulate the BMP signaling pathway by decreasing secretion of MAMPs. The beneficial gut microbiota can stimulate intestinal cells to secrete GIP-1 and GLP-1, which promote the secretion of TGFβ and upregulate BMP/SMADs signaling pathway. Disruption of TGFβ/BMP signaling altered a normally beneficial *Enterobacter* commensal to a pathogenic form [108]. Although calcitriol promotes osteoclast maturation, it strongly inhibits osteoclast lineage commitment by increasing SMAD1 transcription and enhancing BMP-SMAD1 activation [109]. Recombinant human BMP-2 promotes calcium deposition, whereas extracellular calcium stimulates osteogenic differentiation of human adipose-derived stem cells by enhancing expression of BMP-2 [110].

Second, the Wnt signaling pathway may restore bone homeostasis via the gut microbial-dependent signaling network in a positive manner. According to recent studies, *Lactobacillus rhamnosus* GG-induced upregulation of the gut metabolite butyric acid expands the Tregs pool, which subsequently increases the production of the osteogenic Wnt ligand Wnt10b by CD8^+^ T cells to stimulate bone formation [111]. The epithelial Wnt pathways induce antimicrobial peptide expression to provide protection from intestinal infection [112]. Tregs stimulate bone marrow CD8^+^ T cells to produce the Wnt ligand Wnt10b to activate Wnt-dependent osteogenesis. Additionally, PTH promotes bone formation though the Wnt signaling pathway. Butyric acid produced by gut microbiota can aid recovery of bone anabolism induced by PTH, which can then increase the number of Tregs in the bone marrow. Wnt signaling induces osteoblast differentiation and directly suppresses osteoclast differentiation through both canonical (β-catenin) and noncanonical (cAMP/PKA) pathways [113, 114]. Interaction of calcium and gut microbiota regulates bone metabolism by activation of the Wnt/β-catenin signaling pathway [103].

Third, upregulation of OPG expression and downregulation of the RANKL signaling pathway may restore bone homeostasis via the gut microbial-dependent signaling network. Maladjusted gut microbiota interferes with bone immunity by stimulating the release of IL-6 and TNF-α and activating the NF-κB signaling pathway. A lack of sex hormone increases intestinal permeability, which augments the number of Th17 and TNF+ T cells in peripheral blood and raises the concentration of IL-17, potentially inducing osteoclastogenesis by upregulating the RANKL signaling pathway [45]. Therefore, excessive harmful substances produced by dysbiosis cause damage to bone by inhibiting the expression of osteoblast genes and activating that osteoclast genes as well as by activating the NF-κB signaling pathway.

Collectively, a holistic signaling pathway network is proposed based on the gut microbial-dependent signal molecules of bone metabolism and immunity. These pathways have both synergistic and antagonistic effects. Focusing on the signaling pathway network comprehensively rather than paying attention to individual pathways can help us understand gut–bone crosstalk.

## Dietary Interventions

Multiple dietary components potentially affect osteocyte signaling pathways and may have a synergistic effect on bone metabolism and immunity combined with physical activity [115]. Impaired bone growth in animals fed a nutrient poor diet can be improved by either single colonisation of a specific *Lactobacillus* strain or complete reconstruction of the gut microbiota [88]. Dietary intervention in the improvement of bone metabolism via the modulation of gut microbiota that have been proven efficient and accessible [116]. The alteration of gut microbiota can be deemed as a biomarker of bone metabolic activity.

The widely recommended Mediterranean-like diet pattern is a healthy and nutritious diet model for prevention of chronic diseases [117]. The Mediterranean diet elicits favourable microbiota profiles and metabolite production [118]. For example, close adherence to the Mediterranean dietary pattern is associated with lower ratios of Firmicutes to Bacteroidetes and higher faecal SCFA detection [118]. Mediterranean and low-fat diets increase the abundance of *Actinomycetes*, *Bacteroides*, *Prevotella*, *Roseburia*, *Faecalibacterium* and *Ruminococcus* spp. while decreasing that of *Streptococcus* and *Clostridium* spp. [119]. Strict adherence to the Mediterranean and low-fat diets is associated with a lower risk of bone fractures and pain [120] and ameliorates symptomatic forms of osteoarthritis [121] and osteoporosis [122].

Probiotics and prebiotics, bacterial consortium transplantation and faecal microbiota transplantation have been developed to recover the intestinal microecological balance [123]. A six-month supplementation with probiotics was shown to have a favourable effect on increasing bone calcium levels in postmenopausal women during a clinical trial [124]. *Lactobacillus reuteri* regulates dysfunctional gut microbiota and inhibits osteoclast activity through the Wnt10b signaling pathway [125, 126]. Long-term oral administration of *Lactobacillus* and *Bifidobacterium* can increase the activity and quantity of osteoblasts [126, 127]. Besides, prebiotics are metabolised by gut microbiota to produce SCFAs, bile acids and vitamins [5]. Inulin increases the abundance of *Bifidobacterium* spp. and the α-diversity of gut microbiota while decreasing the pH of faeces and increasing calcium absorption [128, 129]. Soluble core fibre has been shown to improve bone strength as a prebiotic in postmenopausal women in a dose-dependent manner [130]. Therefore, with specific diets, probiotics or prebiotics, individualised nutritional plans may be created for people in different clinical environments.

## Conclusion and Perspective

Here, we reviewed how the osteal signaling pathway network is mediated by gut microbiota with a focus on the overall effect. Taking bone metabolism and gut microbiota as a whole will help us obtain a more holistic view on gut microbial functioning and interaction with the host environment and provide more scientific intervention and treatment strategies. However, studies have not sufficiently investigated the interaction between gut microbiota and the pathophysiology of the skeletal system [131]. There are also several key challenges and approaches to improving our understanding of the association between gut microbiota and bone homeostasis. For example, the relatively slow rate of bone turnover makes experimental detection of continuous changes of gut microbiota difficult. Besides, it remains elusive whether the outcomes of the animal experiments and clinical trials are derived from the host that impacted by gut microbiota or gut microbiota *per se*. Research is required to determine whether translocation of metabolites of gut microbiota plays a dominant role in the gut–bone axis, and what is the relative contribution of each metabolite. Thus, the synergy, antagonism and self-management role of the main signaling pathways in the osteal signaling pathway network by gut microbiota merit further study to help make the gut microbiota a promising therapeutic target for treating bone diseases, addressing pitfalls in the current areas that has clinical potential to reduce the societal burden of bone diseases.
